# Local disease risk management practices and barriers to vaccination against peste des petits ruminants among smallholder sheep and goat farmers in northern Nigeria

**DOI:** 10.1016/j.vas.2026.100868

**Published:** 2026-09-17

**Authors:** Ladi Amos Chabiri, Rebecca Paul Weka, Matthew Yakop Gukut, Usman Adamu Rayyanu, Mayowa Peter Olabode, Victoria Isioma Ifende, Thomas Duniya Bidoli, Muhammad-Bashir Bolajoko, Pwaveno H. Bamaiyi, Maryam Muhammad, Marion Bordier, Alexis Delabouglise

**Affiliations:** a Department of Diagnostic Services, National Veterinary Research Institute, Vom, Plateau State, Nigeria; b Department of Veterinary Outreach and Extension Services, National Veterinary Research Institute, Vom, Plateau State, Nigeria; c Veterinary Public Health and Preventive Medicine Department, National Veterinary Research Institute, Vom, Plateau State, Nigeria; d Department of Agricultural Extension and Rural Sociology at Ahmadu Bello University (ABU), Zaria, Kaduna State, Nigeria.; e National Agricultural Extension and Research Liaison Services (NAERLS), Ahmadu Bello University (ABU), Zaria, Kaduna State, Nigeria.; f Department of Veterinary Public Health and Preventive Medicine, Faculty of Veterinary, Medicine, University of Jos, Plateau State, Nigeria; g Bacterial Research Division, National Veterinary Research Institute, Vom, Plateau State, Nigeria; h CIRAD, UMR ASTRE, 34398, Montpellier, France; i ASTRE, Université de Montpellier, CIRAD, INRAE, Montpellier, France

**Keywords:** Animal health services, Disease management, Nigeria, Peste des petits ruminants, Smallholder farming, Small ruminant, Vaccination

## Abstract

**Background and objectives:**

Peste des Petits Ruminants (PPR) remains a major constraint to small ruminant production in Nigeria, threatening rural livelihood and food security, despite the existence of a national control program. Empirical evidence on farmers’ management practices and reasons for low vaccination uptake remains limited. This study aims to explore local management practices, perceptions, and barriers to vaccination among smallholder farmers in Bauchi, Kano and Plateau states.

**Method:**

A socioeconomic study was conducted in 52 villages across the three states, with a total of 104 gender-disaggregated focus group discussions using participatory approaches to elicit participants’ perception of SR farming and management practices of disease outbreak matching the clinical and epidemiological features of PPR. Qualitative data were thematically analysed to identify dominant disease management strategies and perceived vaccination barriers. Spatial comparison of management profiles across study locations was undertaken to assess clustering patterns and contextual variation.

**Results:**

Small ruminants were mostly valued for their contribution to households’ essential expenditures and religious celebrations and diseases were considered the highest threat to these contributions. Four distinct disease management profiles emerged, determined by the use of disease prevention practices and by responses to disease outbreaks with medical treatments or depopulation. Management profiles exhibited spatial clustering across the three states. Ten major obstacles to vaccination were identified, including limited awareness, poor vaccine access, preference for curative care, reliance on indigenous remedies.

**Contributions:**

The findings underscore the need for community-driven vaccination campaigns that consider local knowledge and practices, improve vaccine accessibility, and integrate trusted local health service providers into government-led PPR control programs.

## Highlights

• Characterization of community disease management with participatory approach factor analysis.
• Management of diseases with various prevention methods, medical treatments and depopulation.
• Spatial clustering of disease management behaviour.
• Informal animal health providers are the main source of medicines and vaccines.
• Vaccination of small ruminants impaired by structural and knowledge barriers constrains.

## Introduction

Small ruminants (SR) have a crucial poverty-alleviating role in the in rural areas of Nigeria and other low-income countries, providing meat, milk, manure, and income to poor farmers and pastoralists. Their contributions enhance food security and improve livelihoods (Herrero, 2020; Yusuf et al., 2018) and their adaptability to arid environments and low-input systems makes them particularly suitable in the context of climate change (Jack et al., 2022). Sheep and goats are essential assets in Africa and South Asia, where poverty rates are highest, contributing to food security and employment (Turk, 2013). Beyond their economic role, these animals hold strong cultural and social significance, symbolizing wealth, respect, and community belonging among rural households. In Nigeria, SRs are an integral part of traditional customs and practices, often used as dowries, sacrificial offerings, or gifts during social events such as weddings, naming ceremonies, and religious celebrations (Mustapha et al., 2024). In 2016, goat and sheep meat production in Nigeria was valued at $373.1 million and $73.4 million, respectively (Herrero, 2020). In Nigeria, demand for mutton and goat meat is projected to increase by 216% between 2012 and 2050, reflecting rapid growth in livestock-product consumption and underscoring the need to address productivity constraints, including infectious diseases affecting SR (FAO, 2019).

Peste des Petits Ruminants (PPR), also known as sheep and goat plague, is a highly contagious viral disease affecting SRs (Kumar et al., 2014). It is caused by the PPR virus (PPRV), a member of the Morbillivirus genus in the Paramyxoviridae family (Amarasinghe et al., 2017). The disease is endemic in sub-Saharan Africa, much of the Middle East, and South Asia (Benfield et al., 2023), posing a significant threat to food security and rural livelihoods (Munir, 2013). PPR outbreaks lead to substantial losses due to high mortality, reduced productivity, and trade restrictions (Elsawalhy et al., 2010). It is endemic in Nigeria, endangering smallholder farmers who rely on SRs for subsistence and income (Chukwudi et al., 2025; Umunna et al., 2021). PPR primarily spreads through direct contact between infected and susceptible animals, with respiratory secretions, saliva, feces, and urine serving as the main sources of viral transmission (Munir et al., 2012). Close proximity facilitates aerosol transmission, especially in confined areas (Herzog et al., 2020). Although the virus does not survive long outside the host, contaminated feed, water, and equipment can still contribute to its spread among animals in close contact (Fayyad & Alzuheir, 2024). Human activities, such as the transportation of infected animals over long distances, contribute to its transmission across regions (EFSA & Mancuso, 2025). Managing PPR in smallholder SR systems is a complex challenge entailing technical, socioeconomic, and cultural factors (Serra et al., 2022). There is no specific treatment for PPR; however, supportive care and management of bacterial and parasitic co-infections can help reduce mortality (Abubakar & Irfan, 2014). Early administration of hyperimmune serum to animals before the onset of diarrhoea can lead to recovery in some cases (Kabir et al., 2019).

Recognizing the global impact of PPR, the World Organization for Animal Health (WOAH) and the Food and Agriculture Organization (FAO) launched the Global Strategy for the Control and Eradication of PPR (PPR-GEP), aiming for eradication by 2030 through vaccination and disease surveillance (WOAH & FAO, 2015). Effective vaccination strategies depend on multiple factors, including vaccine availability, affordability, acceptability, and farmer awareness (Wane et al., 2020). Despite the demonstrated efficacy of vaccination, its effective uptake in smallholder farms remains low in Sub-Saharan Africa (Gizaw et al., 2021). Studies indicate that low vaccination coverage in regions such as Uganda results from limited awareness, poor access to veterinary services, and logistical challenges associated with vaccine supply to remote areas (Acosta et al., 2019). In South East Nigeria, inadequate knowledge about PPR vaccination among SR farmers, limited veterinary involvement, and vaccine unavailability at veterinary establishments contribute to poor disease control (Chukwudi et al., 2024). Risk perception is also a critical factor in disease management, as farmers’ understanding of PPR influences their willingness to engage in control practices, including vaccination (Omondi et al., 2022; Wane et al., 2020). Research has shown that farmers in areas with frequent outbreaks are more likely to recognize symptoms and vaccinate their animals compared to others (Benfield et al., 2023). In addition, livestock farmers may attribute disease to spiritual forces, witchcraft, or ancestral displeasure, and therefore prioritise herbal remedies, prayer, and traditional treatments over conventional veterinary medicine (Alhaji & Babalobi, 2015; Suleiman et al., 2018), which affects the implementation of prophylactic and biosecurity measures (Namatovu et al., 2022). Additionally, beliefs about vaccine effectiveness impact adoption rates, with trust in vaccines being higher when they are sourced from reliable providers. Misinformation and previous negative experiences can deter participation in vaccination programs (Ludgate & Adi, 2020; Nuvey et al., 2023). In smallholder farming systems with limited government support, PPR management is largely reliant on local disease control strategies implemented by farmers (Kodo et al., 2025). Studies in Kenya and Senegal reported the common use of quarantine of newly introduced animals, isolation of the sick animals in a separate space and basic sanitation of farm facilities to limit the risk of disease outbreaks in farmer communities, but financial and infrastructural constraints make these measures difficult to sustain (Aboah et al., 2024; Gitonga et al., 2016). Alternatively, farmers may rely on prophylactic treatments with conventional medicines or local remedies (Alhaji & Babalobi, 2015) or may respond to disease outbreaks by adapting animal movements and herd destocking (Delabouglise et al, 2020). Local determinants of the livestock diseases management practices have not been extensively studied. Previous research conducted on livestock farming in low-income contexts identified the degree of commercial orientation of farms as a significant predictor of the animal health management practices (Jemberu et al., 2015), while some authors emphasized on the geographical disparities in veterinary services and local norms (Acosta et al., 2022) and the broader socioeconomic contexts as critical sources of variation in such behaviours (Ebata et al., 2020).

In Nigeria, little is known about how rural communities perceive the risk posed by SR diseases for their livelihood, the way they manage the risk of infectious diseases like PPR and the factors that influence their strategies. Additionally, limited data exist on barriers to effective uptake of vaccination against PPR. The eclectic and complex nature of the perception of livestock and animal health and the management of livestock diseases, as well as the diversity of potential obstacles to vaccine use (Donadeu et al., 2019), justify the use of qualitative or semi-quantitative survey methods for addressing this knowledge gap, as these empirical approaches provide the necessary degree of flexibility and involvement of the survey respondents to identify the different thematic dimensions connected to a given research question (Catley et al., 2012).

This study aimed at exploring the local management practices, disease risk perceptions, and obstacles to access vaccination services to control PPR among smallholder farmers in Northern Nigeria. The specific objectives were to (1) characterize the management practices used by smallholder farmers in response to PPR; (2) identify the obstacles smallholder farmers face in accessing and utilizing PPR vaccines; (3) analyse the relative contribution of geographical proximity and socioeconomic factors on the adoption and sharing of PPR management practices among neighbouring smallholder farmer communities. Addressing these objectives provides complementary perspectives on the overall issue of aligning local disease management practices with the global eradication strategy for PPR.

## Materials and methods

A cross-sectional study was conducted among SR smallholder farmer communities in regions of Nigeria with a known endemic circulation of PPR virus (Mantip et al., 2022), comprising Plateau, Bauchi and Kano States. Data collection was conducted using focus group discussions (FGDs).

### The study area

Plateau, Bauchi and Kano States are in the Northern-Central, North-Eastern and NorthWestern geopolitical zones of the country, respectively, and present distinct characteristics in terms of demography and economy. Nevertheless, the extensive farming of SR is an important part of the rural economy of the three States, and they are similarly affected by PPR, according to previous epidemiological studies (Woma et al., 2016; Rayyanu et al.*,* unpublished results) (Fig. 1).

**Fig. 1:**
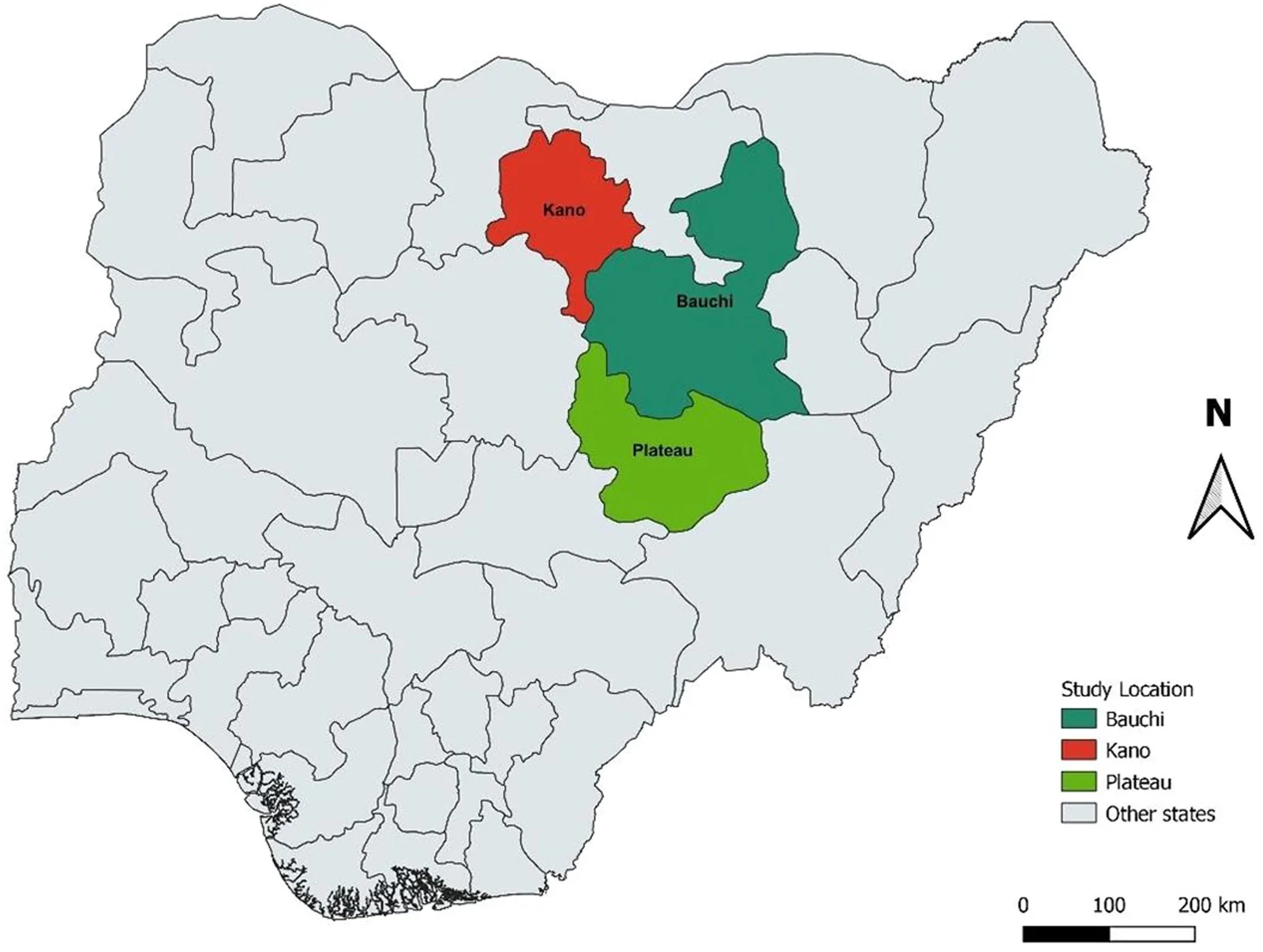
Map of Nigeria indicating the location of the three states where the study was conducted.

Bauchi State is located in the northeastern region of Nigeria and has an estimated population of about 7.5 million people (NBS, 2020). Its economy is largely agrarian, with crop farming and livestock production serving as the primary sources of livelihood for most residents. The state falls within both the Sudan Savannah and Guinea Savannah ecological zones, with a tropical climate, which supports diverse vegetation and agricultural activities (Dantani & Ige, 2024). In 2005, PPR seroprevalence in Bauchi State was reported at 24.7%, reflecting a significant presence of the virus in the region (Luther et al., 2006). Kano State, located in the northwestern part of Nigeria, is the country’s most populous state, with over 14.2 million inhabitants (NBS, 2020). Its economy relies heavily on agriculture and livestock. Situated in the Sudan Savannah zone, Kano experiences a tropical wet and dry climate (Lawal & Lawal, 2017). Livestock production, including cattle, sheep, goats, poultry, and donkeys, plays a vital role in the rural economy, often practiced alongside crop farming but it encounters major challenges such as feed shortages during the dry season, disease outbreaks, and competition among farmers for resources (Ikhuoso et al., 2020). A retrospective study conducted from 2012 to 2016 reported a PPR seroprevalence of 76.88% in goats and 23.12% in sheep (Yahaya et al., 2020). Plateau State, located in Nigeria’s north-central region, within the Guinea Savannah zone, is characterized by its highlands and temperate climate. The state has an estimated population of over 4.4 million people (NBS, 2020). Agriculture is the mainstay of its economy, with the production of potatoes, maize, vegetables, and fruits, alongside significant livestock rearing (Odjugo et al., conference communication). A recent serosurvey in Plateau State showed widespread exposure to PPR, with 85.5% of villages having at least one seropositive flock and an animal-level prevalence of 36.6% (Rayyanu et al., unpublished results). Plateau was one of the states with the highest number of PPR suspicion reports over 2013-2022 (Chabiri et al., 2023).

### Sampling strategy and participant recruitment

The survey targeted the population of smallholder extensive SR farmers of the three states. Initially, 200, 220 and 82 villages were randomly selected respectively in Plateau, Bauchi and Kano states to conduct a serological survey to assess the epidemiological situation of PPR in the three states (Rayyanu et al., unpublished results). A sample of approximately 10% of these villages was selected for interviews with farmers, i.e., 20 villages in Plateau, 22 villages in Bauchi and 10 villages in Kano. The selection of villages was made with the help of the state veterinary services in order to ensure a good balance between the number of seropositive villages for PPR (villages where at least one seropositive animal had been detected with the serological survey) and seronegative villages (villages where no positive animal had been detected), while ensuring adequate geographical coverage in the three states. In each village a group of approximately 10 male participants and a group of approximately 10 female participants was constituted, making a total of 20 participants per village. Participants were recruited with the help of the local authorities, most commonly village heads. All participants were involved in SR farming in a small scale and extensive way. The survey was conducted during the periods July-September 2021, June-September 2022 and April-June 2024 in Plateau, Bauchi and Kano respectively.

### Data collection

FGDs were conducted with the constituted groups of participants using semi-structured interview techniques. Female-only and male-only groups were interviewed by research teams comprising female-only and male-only investigators, respectively. The topics addressed during the interviews included (1) local knowledge of diseases and disease names matching the clinical picture of PPR, (2) socioeconomic context of SR farming, and (3) local management of the disease outbreaks by households. Interactive tools commonly applied in participatory rural appraisals were used for visualization purposes and to collect semi-quantitative metrics (Catley & Alders, 2012). These tools have the advantage of emulating an active involvement of participants, the expression of their perception and opinion on the addressed topics as well as discussions among them. They also allow investigators to cross-check the participants’ answers with the results of the visualization tools, to identify any inconsistency and address potential misunderstandings between the research team and the participants. The research team included at least one facilitator, one note-taker and, when necessary, one translator, in the rare instances where facilitators or the note-takers were not familiar with the language spoken by the participants. Discussions were primarily conducted in Hausa, the regional lingua franca, although in Plateau State participants often switched between Hausa and English.

The first step of the FGD involved identifying the disease most likely to be PPR among the SR diseases known by participants. A method based on participatory epidemiology principles was used, which included listing and describing the SR diseases known by participants that cause significant damage, and evaluating the relative importance of the most common clinical signs associated with these diseases across the five most impactful ones using a matrix scoring technique. The research team chose one of the listed diseases, later referred to as “PPR-like disease”, based on the clinical picture commonly described in the literature for PPR. The adopted methodology is described in detail in (Kodo et al., 2025). The second step of the FGD addressed the following socioeconomic aspects: (1) the reasons for keeping SRs (2) the inputs used for the SR farming activities and their origin and (3) the risks affecting SR farming. The purposes of keeping SRs were listed and classified according to their degree of importance for the livelihood of smallholder farmers using the proportional piling (PP) tool. The listed purposes were written down on a chart and participants were asked to distribute 100 beans across the different items of the list, with the number of beans assigned to each item being in proportion to the importance of the corresponding purpose for the households’ livelihood. The beans were then counted and the number of beans allocated to each purpose were written on the chart. Participants listed the categories of inputs needed for SR farming, and for each of these categories, the sources of the inputs were listed in turn. For example, on category of input was feed, and the potential sources were feed purchases, grazing and leftovers from the household. The categories of inputs and their sources were progressively written down on a separate chart following a flow diagram structure. The risks affecting SR farming were identified by participants and attributed a score according to their perceived severity using PP.

The third step of the FGD addressed the management of PPR-like diseases. Participants were asked to list the measures taken to limit the impact of the PPR-like disease when it was absent (preventive measures) and when it occurred (reactive measures). Preventive and reactive measures were scored separately, according to their perceived importance for the farmers, using PP. During the exercise, participants were asked about their reasons to ascribe a higher importance to some measures compared to others. If the participants mentioned vaccination among the preventive measures, the origins of the vaccines were listed and scored using PP. If vaccination was not mentioned, the facilitator asked participants if they knew about the possibility of vaccinating animals and, if yes, the reason they did not include it in the list of measures.

### Data material

The documents resulting from the interviews consisted in (1) notes taken during the FGDs transcribed in electronic format and translated into English language, including qualitative statements and observations made by participants along the interview process and potential disagreements or debate among them; (2) pictures of charts used for visual tools, including flow diagrams and PPs. The qualitative and semi-quantitative information of interest was extracted from these documents and stored in a single dataset. Each FGD corresponded to a specific observation. Quantitative variables were the PP scores attributed to the following categories of items: (1) purpose of SR farming, (2) risks associated with SR farming, and (3) preventive and (4) reactive actions applied to mitigate the impact of PPR-like diseases on farmers. If an item (purpose, risk, preventive or reactive action) was not mentioned in a group, it was attributed the score 0 for this specific group. After consultation with the research team members, some items were merged since they were considered to be similar despite the use of different terminologies. For example, among the purposes of SR farming, the terms “festivities”, “ceremonies” and “celebration” were considered to refer to a single purpose named “celebration”, and the scores attributed to these purposes were summed up. For preventive actions against PPR-like diseases, one of the mentioned actions was “do nothing”. In case a group of farmers did not mention any particular measure implemented to mitigate the impact of PPR-like disease, this item was attributed the score 100. In order to verify that the count of beans was properly done, PP scores of each category were summed for each FGDs. If the sum was lower than 90 or higher than 110, the observation was considered invalid and withdrawn from the dataset. Categorical variables corresponded to the farming inputs mentioned by participants in the flow diagrams. For example, if the purchase of feed was mentioned by a fraction of the groups, then “feed purchase” was considered as a binary variable taking the value 1 if purchases of feed were mentioned during the FGD and 0 if not. Fragments of text referring directly or indirectly to potential obstacles to vaccination against PPR were also extracted from each interview notes transcripts and stored in a specific table.

### Data analysis

The data was analysed with a mixed methodology combining qualitative and quantitative analytical procedures aimed at addressing specific objectives: (1) contribution of SR to the communities’ livelihood and risk perception were analysed through descriptive statistics of corresponding PP scores; (2) PPR-like disease risk management practices were analysed though descriptive statistics and multiple factorial analysis combined with hierarchical clustering on PP scores in order to identify distinct management profiles among communities; (3) the determinants of these practices were analysed using a multinomial logistic regression model incorporating socioeconomic and spatial factors; (4) obstacles to PPR vaccination were identified through qualitative thematic analysis of FGD reports, allowing the classification of perceived barriers into key themes.

a. Multiple factorial analysis and hierarchical clustering of PPR-like disease management

In order to characterize the communities’ PPR management strategies, PP scores attributed to preventive and reactive measures were first described using simple statistics (frequency of occurrence in FGDs, mean attributed PP scores). A multiple factor analysis was performed on the scores, using the reactive and preventive measures as the two pre-defined groups of quantitative variables (Husson et al., 2011). Multiple factor analysis allows the inclusion of both quantitative variables and qualitative variables. It also allows the contribution of each group (which may include different numbers of variables) to be normalized in order to equalize their maximum influence (inertia) and thus balancing their respective influence on the resulting set of principal components (Bécue-Bertaut & Pagès, 2008; Escofier & Pagès, 1994). Interviewed groups of farmers were then clustered using the Hierarchical Clustering on Principal Components (HCPC) method, which uses the inter-group Euclidean distances measured on the selected principal components. The Ward’s agglomerative method was used to build the hierarchical tree and the optimal number of clusters was determined by evaluating the loss of relative inertia resulting from successive partitions of the tree. The number of clusters corresponding to the maximum loss of inertia was considered as the most appropriate (Murtagh & Legendre, 2014). Clusters of farmer groups are referred to as “disease management profiles” in the remainder of the manuscript.

b. Multinomial logistic regression modelling of PPR-like disease management practices

In order to analyse the relative contribution of the spatial proximity of farmers’ groups and of socioeconomic determinants to the establishment of specific disease management profiles, we built a multinomial principal component logistic regression model of the likelihood of farmer groups to belong to the different clusters. We used as predictor variables the spatial location of groups and socioeconomic variables describing the context of SR farming in the visited villages, including its purpose, the type of inputs used and the risks affecting this activity. To build these socioeconomic variables, we used the results of the visualization tools applied to score the purposes and risks associated with SR and to list the input purchased and sources of medicines for SR farming. A multiple factor analysis was performed to reduce the dimensionality of these four groups of variables to a limited set of principal components. The first principal components of each group of variables explaining together more than 30% of the group variance (hereafter referred to as “socioeconomic components”) were kept for inclusion, as socioeconomic components, in the multinomial logistic model.

Considering *Z_i_* the disease management profile of the group of farmers *i,* then the probability, for the group *i* to belong to cluster *k*, as opposed to any other cluster *h* of the ensemble *H* of clusters is given by the following formula:

$$P\left(Z_{i}=k\right)=\frac{e^{{Y_{k}}_{i}}}{\sum_{h\in H}e^{{Y_{h}}_{i}}}$$

With *Y_k_* a cluster-specific combination of a linear function of the principal components and a spatial autocorrelation term modelled as a bidimensional thin-plate spline (Wood, 2003):

$${Y_{k}}_{i}=\sum_{j\in J}{\beta_{k}}^{j}{X^{j}}_{i}+g_{k}\left(Long_{i},\;Lat_{i}\right)+\varepsilon_{ki}$$

With *X*^j^ the vector of values of the principal component *j* from the set of principal components *J*, β_k_^j^ the linear coefficient of the principal component *j* for cluster *k*, and *g*_k_ the spline transform of the spatial coordinates (longitude L*ong*_i_ and latitude *Lat*_i_ ) for cluster *k*. The restricted maximum likelihood estimation method was applied for spline parameter estimation. The dimensions of the thin-plate spline functions were fitted through a dimension penalization process allowing for a shrinkage of the term to zero (Wood et al., 2017; Wood, 2003). Principal components were standardized and excessive multi-collinearity between covariates was assessed beforehand by estimating their variance inflation factor. A model was calibrated on all groups with complete and valid observations, with the group sex (Male or Female) included as an additional explanatory binary variable. In order to assess the relative contribution of the different factors to the model, separate models were also calibrated including the spatial component only (the spline transform of the spatial coordinates, with no principal component) and the principal components only (with no spatial component). These partial models were compared with the full model using likelihood ratio tests. To assess the sensitivity of the model to the number of selected principal component, we performed the exact same modelling approach using only the first principal components of each group of variables, so four in total.

c. Thematic analysis on barriers to PPR-like disease vaccination

The identification of obstacles to PPR vaccination, from the viewpoint of farmers, was performed with a targeted thematic analysis aimed at identifying, in the interview notes, any potential obstacles to the vaccination of SRs against PPR mentioned by the FGD participants and classifying them into themes. Meaning units, that is information or judgements expressed in FGDs, were attributed specific information codes. Information codes were then grouped into themes. Identified themes corresponded to specific factors reducing the likelihood of vaccinating SRs against PPR. Each theme was linked to the number of interviews it was extracted from, disaggregated by state (Bauchi, Kano and Plateau) and gender.

### Computing material

Data entry and thematic analysis were performed with Microsoft Excel. All quantitative analysis were performed with R version 4 (*R: A Language and Environment for Statistical Computing*, n.d.). Factor analysis and clustering of farmer groups, multinomial logistic model and graphical representations were done with the FactoMineR, mgcv and ggplot2 packages respectively.

## Results

A total of 104 FGDs were conducted, involving a total of 1065 participants (554 females and 511 males) with 52 male-only and 52 female-only groups, and 44, 20, and 40 groups in Bauchi, Kano and Plateau State respectively. Three and two groups had missing or invalid PP scores for risks affecting SR farming and reactive actions against PPR-like diseases respectively. All other observations (n=99) were considered valid.

A total of twenty-seven different purposes of SR farming were reported across the three States. The most commonly reported purposes were payment of medical bills (*n* = 89), payment of school fees (*n* = 83), celebrations (*n* = 82), production of manure (*n* = 69), purchase of food for the household (*n* = 58), and home consumption of animal products (*n* = 53) (Fig. 2A). Celebrations received the highest average PP score, followed by payment of medical bills, school fees and food purchase, income generation and manure production. While being frequently cited, home consumption of animal products was assigned a relatively low average PP score compared to other common purposes (Fig. 2A). Twenty-four different risks were identified. Only four risks were cited by more than half of the groups: animal diseases (*n* = 99), animal theft (*n* = 99), accidents (*n* = 70), and conflicts with neighbours (*n* = 66). These were followed by predation (*n* = 48), animals going missing (*n* = 29), and feed scarcity (*n* = 25) (Fig. 2B). Animal diseases and theft received the highest scores, followed by conflicts with neighbours and accidents. Predation, animals going missing, and feed scarcity, although less frequently reported, were also attributed a relatively high PP score (>4) (Fig. 2B). Reported sources of animal health products, ranked by decreasing order of frequency, included local animal health providers (*n* = 66), market vendors (*n* = 38), veterinarians (*n* = 29), locally available medicinal products (*n* = 23), and chemists (*n* = 8) (Fig. 2C). Animal health providers are considered by farmers as local knowledgeable people who can provide support to farmers in the management of animal diseases, without necessarily having a proper degree or training certification. The most commonly reported expenditures were the purchase of young animals (*n* = 88) and feed (*n* = 81), while only a minority of groups reported expenditures on hired labor (*n* = 19) (Fig. 2D). Six principal components were extracted from the Multiple factor analysis, corresponding to farmers’ tendencies to (1) keep SR for the purpose of paying daily expenses, including medical bills, school fee and food, as compared to other purposes, (2) keep SR for income generation as compared to celebrations, (3) attribute a high severity to risks of SR theft as compared to SR diseases, (4) obtain SR medicines from veterinarians rather than animal health providers, (5) purchase SR medicines from vendors or at market rather than using local products found in the environment, and (6) engage expenditures in feed purchase and farm worker wages.

**Fig. 2:**
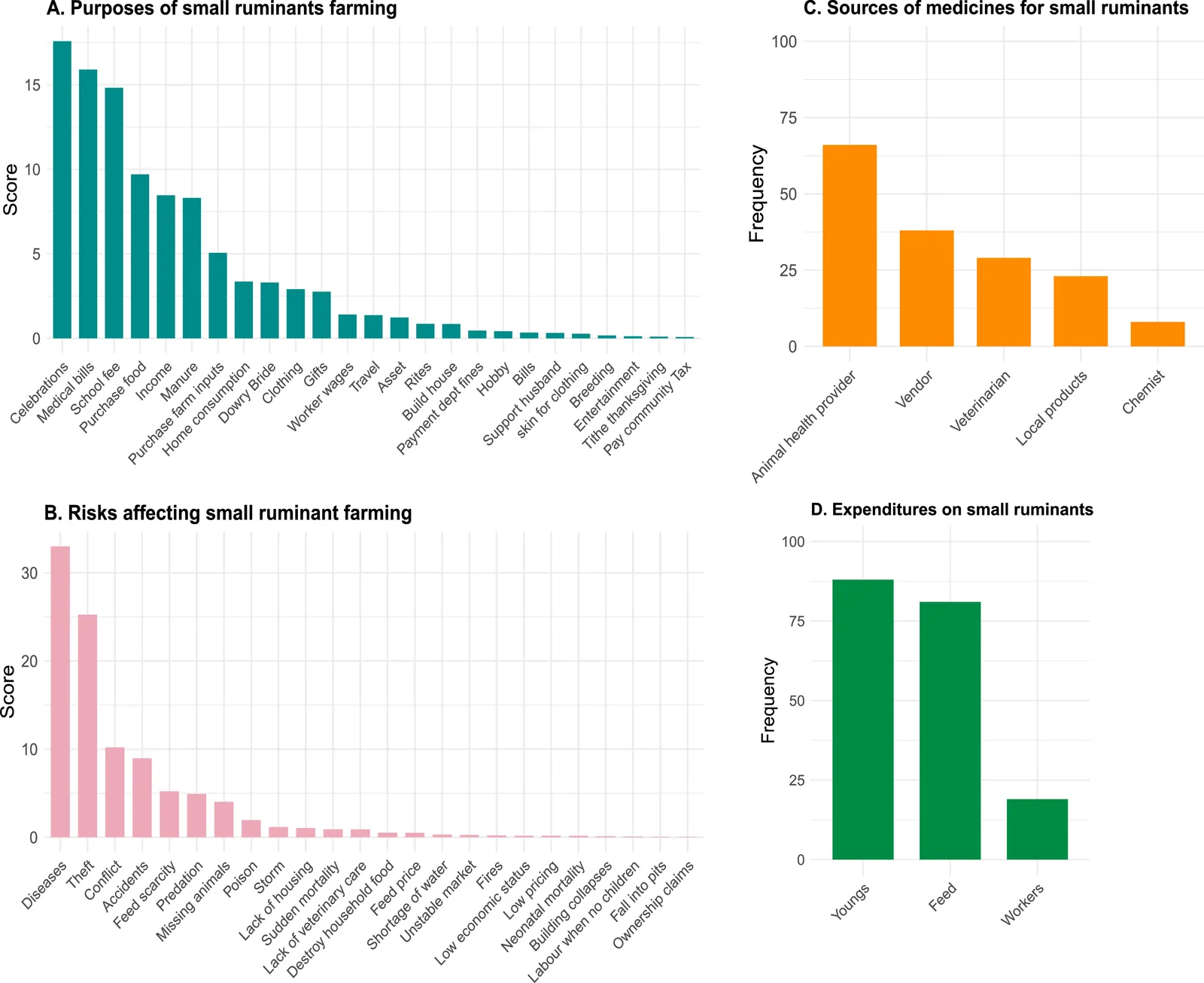
Barplot representation of different results from the focus group discussions conducted with study participants: average proportional piling scores attributed to (A) purposes and (B) risks associated with SR farming, and frequency of mentions of (C) sources of medicines used on SRs by participants and (D) expenditures in farm inputs.

Twenty-eight groups of participants across the 3 states reported no specific measures taken in anticipation of PPR-like disease outbreaks. The other groups reported 15 different measures potentially applied. The most commonly reported measures were prophylactic treatments either with conventional or local medicines (*n* = 44); vaccination (*n* = 34); isolation (i.e. restricting movements of animals) (*n* = 27), and sanitation (i.e. hygiene, cleaning of the facilities) (*n* = 23). These measures were also given the highest scores (Fig. 3A). Other less frequent measures were adequate feeding (*n* = 10) and preventive depopulation (i.e. the sale or slaughter of animals in anticipation of a disease outbreak to limit the anticipated losses) (*n* = 7). The other measures were reported three times at most. Among the 34 groups who reported vaccination as a preventive measure, 29 were able to list their sources of vaccines, which included the animal health provider (*n* = 23), the market, veterinary store or chemist (*n* = 7), the veterinary hospital (*n* = 6) or the government (*n* = 1). All groups reported at least one reactive measure applied in the face of a PPR-like disease. Ten different measures were reported, the two predominant ones being medical treatment of sick animals in an attempt to heal them (*n* = 96) and the depopulation of herds (*n* = 86) (Fig. 3B). Medical treatments were performed either with conventional medicines or with local products found in the environment (for example extracts of Mahogany trees). Conventional medicines were obtained from local animal health providers, market vendors, veterinary stores, veterinary hospitals, or human pharmacies. Depopulation meant the sale at a reduced price, or the slaughter or gift of, generally, the sick animals before their death, but also, in some case, of clinically healthy animals who were likely to become sick in their turn. No sale of dead animals was reported and the consumption of dead animals was reported by only one group in Plateau. The actors who purchased the animals during PPR-like disease outbreaks included traders, middlemen, marker vendors, butchers, neighbours, relatives or acquaintances. The depopulation strategy was motivated by the willingness to limit the loss of income resulting from the death of affected animals. Selling healthy animals before they become sick was further motivated by the loss of economic value of animals displaying clinical signs of a disease. Other less common reactive measures included isolation of sick animals from the healthy ones (*n* = 22), movement restrictions (i.e. limiting the movements of healthy animals to avoid their infection) (*n* = 7), public awareness (i.e. spreading the information on the disease outbreak to other farmers so that they take preventive measures) (*n* = 7), and sanitation (*n* = 5). The other measures were reported 3 times at most.

**Fig. 3:**
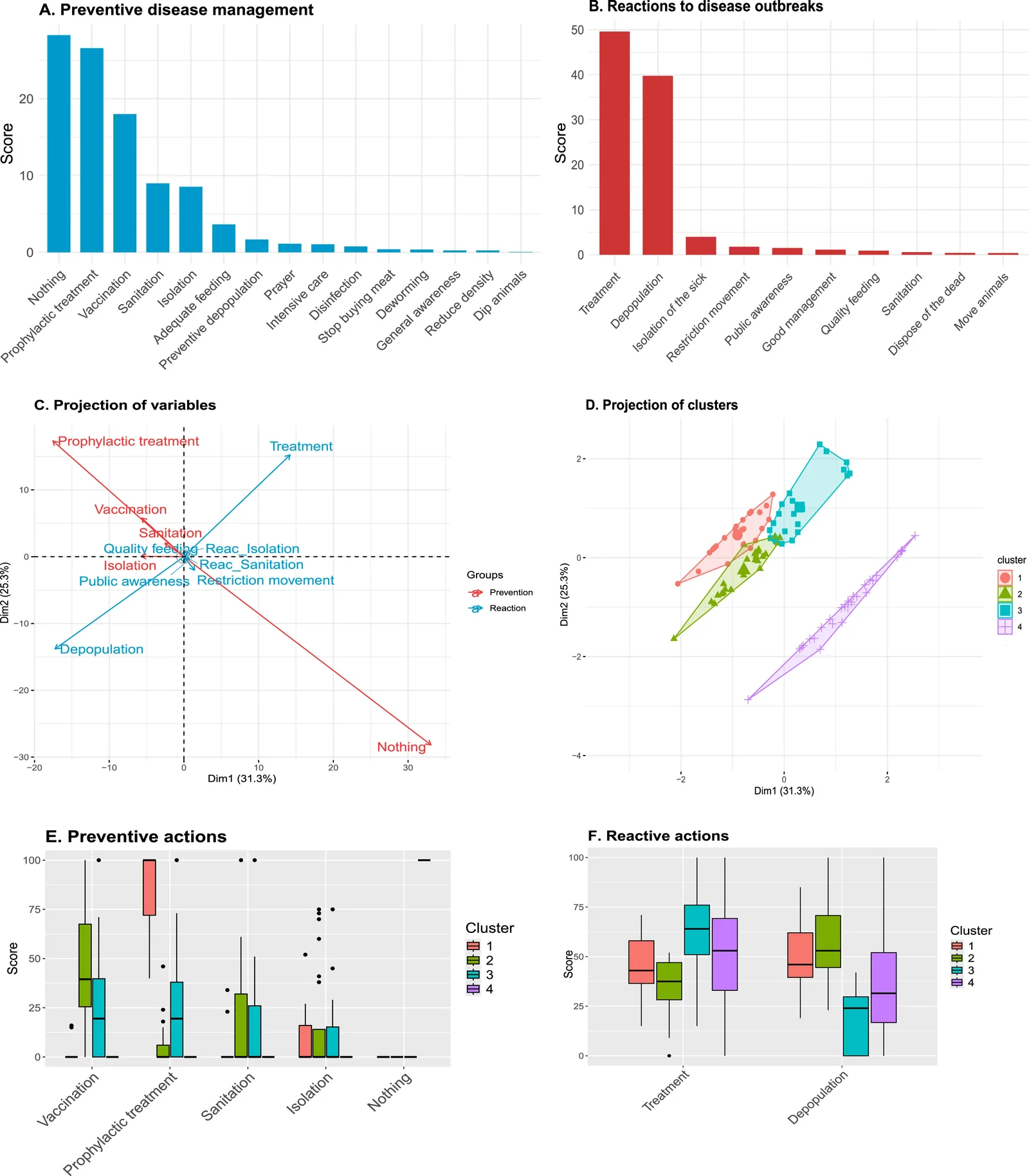
Description of the disease management profiles established among the interviewed groups of farmers. **Top:** average proportional piling scores attributed to (A) preventive and (B) reactive disease management measures. **Middle:** projection of (C) the scores of the main preventive and reactive disease management measures and of (D) the clusters of farmers groups on the two first dimension established with the multiple factors analysis. **Bottom:** boxplot representation of the distribution of the scores of the main (E) preventive and (F) reactive disease management measures disaggregated by clusters.

According to the results of the multiple factor analysis performed on the PP scores attributed to preventive and reactive measures, groups of farmers could be differentiated based on the importance they attributed (1) to depopulation as opposed to medical treatments to respond to PPR-like disease outbreaks, and, (2) to preventive actions taken in anticipation of the PPR-like disease outbreaks, primarily prophylactic treatments, as opposed to doing nothing (Fig. 3C). Clustering of farmer groups was performed using the first three components of the MFA, which together explained more than 70% of the total variance. While the first two components were retained for interpretation and visualization, the third component— capturing additional residual variability among preventive and reactive practices—was included to improve cluster discrimination and stability. Similar results were obtained when using the first four components. Four clusters were identified, corresponding to distinct profiles of PPR-like disease management (Fig. 3D): cluster 1, characterized by prevention with prophylactic treatments and reaction with a mix of depopulation and treatment; cluster 2, characterized by prevention with vaccination and sanitation, and reaction with depopulation rather than treatment; cluster 3 characterized by diversified preventive practices and reaction with treatment rather than depopulation; cluster 4 characterized by an absence of prevention, and reaction with a mix of depopulation and treatment (Fig. 3E and F).

The results of the multinomial principal component logistic regression model fitted to the valid FGD observations (*n* = 99) are displayed in the Table 1. The six socioeconomic components identified with the multiple factor analysis performed on the socioeconomic variables were included as linear terms in the model. Cluster 2 membership corresponded to the baseline situation in our models. The full model, incorporating both principal components and the spatial autocorrelation term, explained 20.2% of the observation deviance. Its goodness-of-fit was significantly higher than the one of the model with the principal components only (likelihood ratio test: *p*-value < 0.01), suggesting a significant effect of geographical proximity on the disease management profile of farmer groups. Spatial terms were fitted for cluster 4 and cluster 1 membership prediction, and the spatial effect was significant for cluster 4 membership prediction. No spatial effect was fitted for cluster 3 membership prediction. Visually, the groups of clusters 1 and 4 tended to be concentrated in the northwestern and northeastern part of the study area respectively, while groups of clusters 2 and 3 were mostly located in its southern part (Fig. 4). By contrast, the full model did not perform significantly better than the model incorporating only the spatial autocorrelation term (see results of the likelihood ratio tests in Table 1). One principal component was found to be significantly associated with cluster membership in the full model, namely the importance attributed to SR farming for income generation as opposed to celebrations was negatively associated with membership in cluster 1 (prevention with prophylactic treatments). Finally male-only groups were significantly more likely than female-only groups to belong to cluster 4 (absence of disease prevention practices). In order to assess the sensitivity of our modelling approach to the number of selected principal components, we implemented the same procedure using only four principal components in the full model, i.e. the first principal components of each group of variables. The results are displayed in **supplementary Table S1**. Again, the full model performed significantly better, in terms of goodness-of-fit, than the model with the principal components only (likelihood ratio test: *p*-value < 0.01), but not better than the model incorporating only the spatial autocorrelation term. One major difference, with this reduced model, was that, in the absence of the principal component indicating an importance attributed to SR farming for income generation as opposed to celebrations, cluster 1 membership was significantly correlated with the spatial spline component but was not significantly correlated with any principal component.

**Table 1:** Results of the statistical models performed to predict the allocation of female-only and male-only groups to disease management profiles. Cells highlighted in gray color indicate statistical significance (*p* value <0.05).

Clusters Total number of observations *n* = 99	Baseline is cluster 2: prevention with vaccination and sanitation, reaction with depopulation rather than treatment (*n* = 26)								
	Cluster 3: diversified preventive practices, reaction with treatment rather than depopulation (*n* = 22)			Cluster 4: absence of prevention, reaction with a mix of depopulation and treatment (*n* = 28)			Cluster 1: prevention with prophylactic treatments, reaction with a mix of depopulation and treatment (*n* = 23)		
Parameter	Coef*	CI*	PV*	Coef	CI	PV	Coef	CI	PV
Purpose of SR : priority to household expenses	0.33	−0.3 to 0.96	0.3	0.76	−0.24 to 1.76	0.14	0.47	−0.39 to 1.33	0.28
Purpose of SR : income rather than celebrations	−0.23	−0.95 to 0.49	0.53	−0.81	−1.75 to 0.14	0.09	-1.21	−2.05 to -0.37	<0.01
Risk: theft rather than diseases	−0.07	−0.7 to 0.55	0.82	−0.02	−0.72 to 0.69	0.96	0.15	−0.55 to 0.85	0.67
Medicines: provided by veterinarian rather than animal health provider	−0.07	−0.7 to 0.57	0.84	−0.15	−0.89 to 0.58	0.68	−0.42	−1.15 to 0.3	0.25
Medicines: purchase from vendors rather than use of local products	0.22	−0.36 to 0.81	0.46	0.12	−0.58 to 0.82	0.73	0.47	−0.21 to 1.15	0.18
Inputs: expenditure in feed purchase and worker wages	0.31	−0.31 to 0.94	0.32	−0.17	−0.93 to 0.58	0.65	0.4	−0.26 to 1.05	0.23
Group gender : Male	0.62	−0.69 to 1.93	0.35	1.73	0.26 to 3.19	0.02	0.95	−0.45 to 2.35	0.18

Spatial thin plate spline (estimated degree of freedom)	0	NA	0.84	1.88	NA	<0.01	0.19	NA	0.28
Percentage of explained deviance – full model	20.2%								
Percentage of explained deviance – principal component regression and gender only	14.4% (likelihood ratio test : *p*- value <0.01)								
Percentage of explained deviance – spatial spline and gender only	15.1% (likelihood ratio test : *p*-value = 0.58)								

⁎ Coef: model coefficient; CI: 95% confidence interval of the model coefficient; PV: *p*-value

**Fig. 4:**
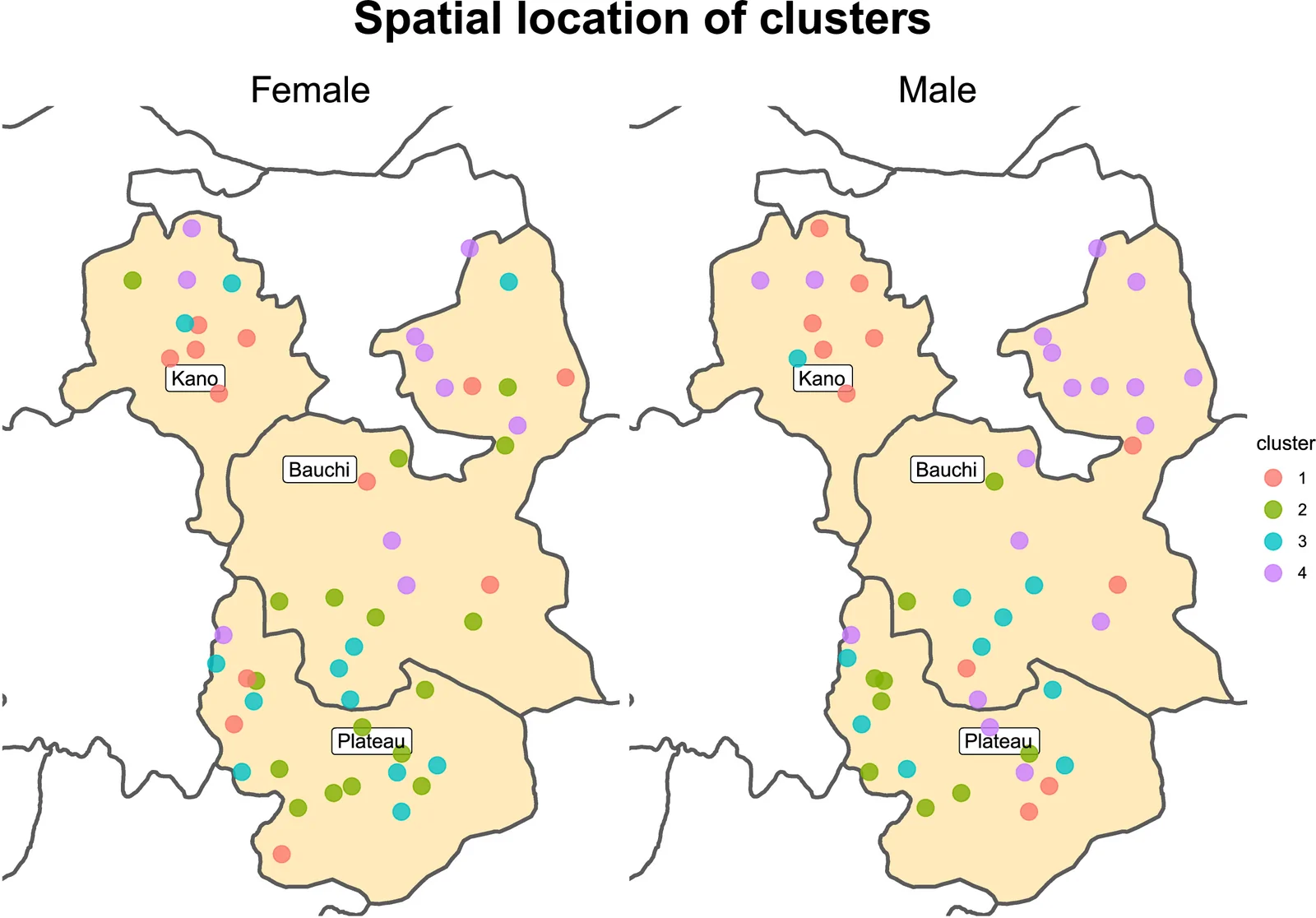
Spatial distribution of female-only and male-only groups belonging to the different clusters in the three study states of Nigeria (Bauchi, Kano and Plateau). Cluster 1: prevention with treatments, depopulation and treatments; cluster 2: prevention with vaccination and sanitation, depopulation rather than treatment; cluster 3: mixed practices of prevention, treatments rather than depopulation; 4: no prevention, depopulation and treatments.

Ten distinct themes corresponding to obstacles to vaccination or to inequalities in vaccination administration (hereafter referred to as “gaps”) were extracted from the analysis of the FGD interview notes (Table 2). Gap 1 referred to a complete absence of preventive attitude expressed by the interviewed groups, with a management strategy of PPR-like diseases purely focused on reaction to disease outbreaks when they occur (*n* = 13). It was observed in groups present in all three states although it was most frequent in Bauchi. Gap 2 referred to the use of other disease management strategies, mentioned as an alternative to vaccination (*n* = 29). It included the use of local products found in the environment as curative or prophylactic medicines. These local treatments were highly diverse in nature. One group, for example, reported grinding neem tree to powder, and mixing it with potash and feed it to the animals. Another one reported grinding leaves of baobab tree and soak it in the animals’ drinking water. Several groups reported mixing grinded mahogany leaves with the animal feed or even burn it so that the smoke sanitizes the animals’ environment. Farmer groups also frequently reported the use of antibiotics, including Septrin, Flagyl and Tetracycline. Gap 3 referred to a limited or total absent knowledge on vaccination, or a confusion between vaccination and treatments (*n* = 18). Gap 4 referred to a divergence between awareness and practice, the concerned groups reporting an absence of implementation of vaccination despite awareness (*n* = 5). Gap 5 referred to a priority given to treatments of diseases when they occur as opposed to their prevention, driven by the belief that treatments of sick animals are sufficient to manage outbreaks without the need of investing in disease prevention (*n* = 2). Gap 6 referred to a perception of SR diseases as inevitable and any attempts at preventing them as useless (*n* = 2). The two groups referred to religious beliefs to justify this perception, by characterizing diseases as “acts of gods”. Gap 7 referred to the anticipation of negative effects of vaccines (*n* = 1). In the concerned group, one woman reported having administered vaccines provided by a government representative once and having subsequently lost all her goats. Gap 8 referred to difficulties in accessing vaccines in areas deprived of animal health services or unusually visited by government officials or animal health professionals (*n* = 2). Gap 9 referred to the prioritization of the most valuable animals for vaccination (*n* = 1). The concerned group of participants reported that vaccination was done on sheep only, because their economic value was considered higher than the one of goats. Gap 10 referred to the practice of vaccination, or other prophylactic measures, in a reactive rather than preventive way (*n* = 8). Participants in those groups reported not using vaccination in a routine way but rather called an animal health provider when disease outbreaks occurred nearby to implement a preventive action, which could include the administration of vaccines.

**Table 2:** Distribution of the number of occurrences of themes corresponding to potential obstacles to vaccination against PPR or inequalities in the practice of vaccination against PPR (referred to as “gaps”) across states and genders. The number of FGDs from which the themes were extracted are displayed in the columns.

Identified barrier to vaccination	Female-only groups			Male-only groups			Total
	Bauchi	Plateau	Kano	Bauchi	Plateau	Kano	
No prevention at all	3	0	1	8	1	0	13
Use of other preventive strategies	3	6	5	4	5	6	29
Limited knowledge on vaccination	3	2	5	6	0	2	18
Gap between knowledge and practice	2	1	1	1	0	0	5
Treatment rather than prevention	0	1	0	1	0	0	2
Helplessness: disease are inevitable	1	0	0	1	0	0	2
Perceived negative effect of vaccines	0	1	0	0	0	0	1
Limited access to vaccines	1	0	0	0	1	0	2
Economic consideration: sheep more valuable than goats	0	1	0	0	0	0	1
Reactive vaccination	0	0	4	1	0	3	8

## Discussion

The findings highlight the multifaceted role played by SR farming in sustaining rural livelihoods in Nigeria, aligning with earlier studies (Jack et al., 2022; Yusuf et al., 2018). Sheep and goats serve as multifunctional assets, contributing both economically and socioculturally to household well-being. Their high ranking as sources of funds for medical bills, school fees, and ceremonial expenses, including sacrifices, highlights their importance as “livestock savings”, a store of value that can be readily liquidated to meet urgent needs. Similar patterns have been reported in other parts of Sub-Saharan Africa, where SRs serve as buffers against economic shocks (FAO, 2018; Kotchofa et al., 2021). Although home consumption was frequently mentioned, its relatively low PP score suggests that SR keeping is largely market-oriented and that its commercial and social value outweighs its direct contribution to household nutrition. Finally, the attribution of the highest mean PP score to celebrations is consistent with the importance of sheep for Muslim celebrations. SR diseases constitute the greatest perceived risk to SR farming, a finding that resonates with the endemic nature and high prevalence of PPR and other infectious diseases in the study area (Bello et al., 2016; Yahaya et al., 2020; Rayyanu et al., unpublished results). For these reasons, farmers place a high value on disease risk reduction, a context favourable to the adoption of vaccination.

Reported preventive practices markedly departed from the official recommendations. Less than one third of FGDs reported routine vaccination, a finding consistent with the low vaccination coverage reported in other Nigerian studies (Chukwudi et al., 2024). This proportion might actually be an overestimate of the true fraction of communities benefiting from PPR vaccination. Indeed, a fraction of the FGDs participants who reported the use of vaccination against the PPR-like disease might have actually referred either to vaccination against another infectious disease (e.g. sheep and goat pox) or to another injectable medicine (e.g. antibiotics). The low vaccination coverage highlights persistent structural barriers to vaccination, including low awareness of vaccines, limited vaccine availability and poor access to veterinary services as well as substitution by other forms of disease management. When preventive measures were reported, prophylactic treatments with conventional or indigenous medicines were most common but farmers reported a high diversity of alternative strategies that included a change in animal feeding, a regulation of animal movement and herd destocking. In the current situation, the low vaccination uptake is most likely attributable to (1) insufficiencies in the formal vaccine supply, (2) limited accessibility to veterinary services at community level, or (3) gaps in the diffusion of vaccine-related information to communities. The cost of vaccination was never explicitly mentioned as an obstacle, which, for most respondent, may be a direct consequence of their lack of awareness or access to vaccination, precluding any consideration to its cost. However, it may also indicate that farmers already familiar with vaccination are sufficiently conscious of its economic value and consider it affordable. Limited understanding on vaccination and its preventive role emerged as another key issue. Although thirty-four groups reported the use of vaccination as a preventive measure, some of these groups reported vaccinating when an outbreak was occurring in neighbouring farms, a practice that can be considered as “reactive” vaccination. Additionally, confusions between vaccination and treatments were frequently discovered during the discussions, leading investigators to re-classify what respondent named “vaccination” as treatments used as prophylaxis. It is therefore possible that a significant fraction of the delivered vaccines might be used in an inappropriate way.

Local animal health providers served as the primary source of vaccines and medicines. The limited reference to government veterinary services indicates a weak direct interface between public veterinary institutions and smallholder farmers in PPR risk management. Similar structural obstacles have been documented in other low-income livestock systems, where public sector limitations compels smallholders to rely heavily on informal networks and private service providers (Acosta et al., 2022; Wane et al., 2020). In Nigeria, this gap could be adequately filled by strengthening the involvement of local animal health providers in disease control programs and vaccine delivery, provided they receive an appropriate training and supervision (Bugeza et al., 2017) In this regard, the training, certification and supervision of community animal health workers (CAHWs) appears to be a promising way forward (Acosta et al., 2019; Donadeu et al., 2019). Evidences suggest that well-trained CAHWs enhance disease detection and reporting, but their financial sustainability is undermined by farmers' low willingness to pay for their services (Bugeza et al., 2017). Despite these barriers, community-led vaccination initiatives piloted by CAHWs or community leaders have been reported to successfully increase vaccination rates (Dione et al., 2019). In the context of Nigeria, establishing linkages between the public sector and the local providers of animal health services would ensure that the latter outsource their vaccines from official channels rather than from the informal sector, limiting the risk of use of poor quality or counterfeit vaccines.

While only a fraction of households reported preventive strategies, all of them reported at least one reactive form of PPR-like disease management. Most commonly, households resorted to medical treatments, while the other major response was depopulation - selling off potentially infected animals to salvage financial value before mortality occurred. Although economically rational, this practice poses significant epidemiological risks by facilitating disease spread, potentially over long distance, especially through live animal trade in markets, where infected subjects can contaminate a large number of in-contact animals (Munir, 2013; WOAH & FAO, 2015). The coexistence of conventional veterinary medicine and ethnoveterinary remedies, including plant-based therapies has been reported in other studies in Nigeria and other settings (Adeyeye et al., 2021, Namatovu et al., 2022). This duality can enhance resilience and underscores the need for integrated control strategies that respect local practices while promoting evidence-based interventions and warning against potential externalities of inappropriate medical treatments. Indeed, the commonly reported use of antibiotics to treat PPR-like diseases may increase the risk of antimicrobial resistance development in bacterial pathogens (No et al., 2018).

The results of the statistical model suggest that spatial proximity has a major influence on the adoption of preventive disease management practices, as evidenced by the strong effect of the spatial component terms on the likelihood of absence of disease prevention practices (cluster 4). This would imply the existence of a social diffusion of management strategies between neighbouring communities, facilitated by imitation and informal knowledge sharing. This result supports the use of community-led extension strategies, where interventions introduced in strategically selected “influential” communities, could organically spread to surrounding areas (Dione et al., 2019; Gizaw et al., 2021). Targeted awareness campaigns should therefore leverage spatial dynamics by focusing on central or socially connected communities. Such an approach would enhance the efficiency and outreach potential of vaccination programs, improving coverage across wider geographic clusters while minimizing resource spending. Nevertheless, the effect of shared geographical area may still be confounded, in this analysis, with other factors, like agro-ecological conditions, level of epidemiological risk and quality of veterinary services, which our study does not fully capture. The influence of spatial proximity would therefore need to be confirmed by additional studies. In addition, male groups were more likely to report a complete absence of disease prevention practice (cluster 4). This result is consistent with a gendered division of labour related to SR care reported in other studies (Kariuki et al., 2022), a higher involvement of women in the daily car of SR as compared to men presumably resulting in a comparatively more complete knowledge of behaviours aimed at mitigating disease impacts at herd-level. Finally, households valuing SR primarily for religious celebrations seemingly were more likely to belong to cluster 4, i.e. to implement a medical prophylaxis with locally-sourced medicines. This result would need to be confirmed with additional studies, since this effect is confounded with the spatial proximity, the communities giving the highest score to celebrations being clustered in the state of Kano, in the north-western part of the study area. It is possible that farmers readily invest particular efforts in maintaining animals destinated to religious sacrifice in good health, while the limited revenues derived from SR compel them to use medicines sourced from their direct environment rather than purchased veterinary products.

Current management strategies are heavily reliant on informal service providers and reactive coping measures. To advance toward the 2030 PPR eradication goal, several priorities emerge, including (1) strengthening linkages between government veterinary services, local animal health providers, and farming communities; (2) expanding training and capacity building of CAHWs; (3) implementing tailored awareness campaigns that integrate trusted local practices while promoting preventive vaccination and warning against negative externalities of some specific disease management strategies (e.g. rapid sales, antibiotic treatments); (4) leveraging social diffusion to shift behavior from reactive to preventive approaches. By addressing these gaps, Nigeria can enhance vaccination uptake, strengthen community trust, and build more resilient SR production systems.

Our study has some limitations. It describes the management of cases of SR diseases that, according to the clinical description provided by farmers, are the most likely to correspond to cases of PPR, but are not actual cases of PPR confirmed through laboratory tests. In a region where PPR is endemic, with strong evidences of high PPR prevalence, it is reasonable to assume that the described management practices are, indeed, the ones implemented in response to PPR or to the risk PPR outbreaks, even if those management practices may be applied on a wider range of infectious diseases with similar epidemiological features. The selected villages orginated from an original sample of villages targeted by a serosurvey established with the help of the state veterinary services and FGD participants were recruited with the help of the local authorities, most commonly village heads, who identified people considered as SR farmers. Although we took care of incorporating villages with different features in terms of geographical location and serological status for PPR, our sample cannot really be considered as random and we cannot exclude the influence of sample biases on the study results. We also cannot completely exclude that the implementation of the study in different years (2021, 2022 and 2024 in Plateau, Bauchi and Kano respectively) may also have contributed to some of the observed geographical differences in the study results, which may partly reflect temporal changes in PPR occurrence, vaccination activities and access to veterinary services. Despite the use participatory methods aimed at eliciting the free expression of the participants’ perception and opinions and open discussions among them, we cannot exclude the influence of social desirability biases in their responses. Although PPR outbreaks are frequent in the region, we cannot exclude that recall biases may have affected the reports in the some of the communities where no outbreaks had been observed for an extended period.

## Conclusion

This study underscores the diversity of the roles played by SR farming in Nigeria and the importance attributed to diseases risks posed by PPR by smallholder farmers. Despite recognizing PPR as a major risk, preventive measures remain limited due inadequate vaccine availability, poor access to animal health services, knowledge gaps and misconceptions, inefficient use and reliance on alternative risk-coping practices. The findings also suggest that social diffusion between communities favoured by geographical proximity influences the adoption of disease management practices. Strengthening community-led extension systems, improving vaccine delivery mechanisms, and integrating local knowledge into formal disease control programs appear to be promising solutions to improve vaccination coverage against PPR and expend and securize the services provided by SR to rural populations.

## Ethics declaration

Written informed consent to take part in the study and to publish the article has been obtained from all participants or their legal representatives. The privacy rights of participants have been observed.

This study was performed in compliance with relevant laws, regulatory frameworks and guidelines where the research took place. This study was conducted in accordance with General Data Protection Regulation (GDPR) (2016/679). This study was approved by the National Veterinary Research Institute. (Approval No. NVRI/AEC/02/113A/22).

The study was implemented in accordance with the General Data Protection Regulation (GDPR) (2016/679) of the European Union concerning the collection and use of personal data. In the study areas, permission was obtained from all relevant authorities before field data collection. Written informed consent was obtained from study participants and was documented with a written signed consent form, prior to their involvement in the FGDs. The collected data was only shared among the researchers directly involved in the data verification, cleaning, processing and analysis. The National Veterinary Research Institute Committee provided ethical clearance for the study, including the involvement of collection of data from human participants, with permission number NVRI/AEC/02/113A/22.

## Funding

The research was funded by a grant from European Commission (Development Cooperation Instruments) awarded to the project ‘EU Support to Livestock Disease Surveillance Knowledge Integration’ (LIDISKI) (FOOD/2019/410-957) within the framework of the Development of Smart Innovation through Research in Agriculture (DeSIRa) programme.

## CRediT authorship contribution statement

**Ladi Amos Chabiri:** Writing – original draft, Methodology, Investigation, Formal analysis, Data curation. **Rebecca Paul Weka:** Writing – review & editing, Validation, Supervision, Investigation. **Matthew Yakop Gukut:** Investigation. **Usman Adamu Rayyanu:** Investigation, Data curation. **Mayowa Peter Olabode:** Investigation. **Victoria Isioma Ifende:** Writing – review & editing, Validation, Supervision, Project administration, Methodology. **Thomas Duniya Bidoli:** Project administration, Methodology, Investigation, Conceptualization. **Muhammad-Bashir Bolajoko:** Writing – review & editing, Project administration. **Pwaveno H. Bamaiyi:** Writing – review & editing, Supervision. **Maryam Muhammad:** Project administration. **Marion Bordier:** Writing – review & editing, Validation, Project administration, Conceptualization. **Alexis Delabouglise:** Writing – review & editing, Writing – original draft, Visualization, Supervision, Project administration, Methodology, Formal analysis, Data curation, Conceptualization.

## Declaration of competing interest

The authors declare that they have no known competing financial interests or personal relationships that could have appeared to influence the work reported in this paper.
