# Supplementary material for: Local disease risk management practices and barriers to vaccination against peste des petits ruminants among smallholder sheep and goat farmers in northern Nigeria
Source: Vet Anim Sci. 2026 Sep 17;34:100868. doi: 10.1016/j.vas.2026.100868 (PMC13635253; doi:10.1016/j.vas.2026.100868)
Supplement: Supplementary file 1 [file mmc1.pdf]

Table S1. Results of the statistical models performed to predict the allocation of female-only and male-only groups to disease management profiles, using a reduced set of principal components (four instead of six). Cells highlighted in gray color indicate statistical significance (p value <0.05)

Clusters<br>Total number of observations n=99	Baseline is cluster 2: prevention with vaccination and sanitation, reaction with depopulation rather than treatment (n=26)								
	Cluster 3: diversified preventive practices, reaction with treatment rather than depopulation (n=22)			Cluster 4: absence of prevention, reaction with a mix of depopulation and treatment (n=28)			Cluster 1: prevention with prophylactic treatments, reaction with a mix of depopulation and treatment (n=23)		
Parameter	Coef*	CI*	PV*	Coef	CI	PV	Coef	CI	PV
Purpose of SR : priority to household expenses	0.37	-0.2 - 0.94	0.2	0.84	-0.01 - 1.7	0.05	0.46	-0.2 - 1.11	0.17
Risk: theft rather than diseases	-0.14	-0.73 - 0.46	0.65	-0.14	-0.8 - 0.53	0.69	-0.05	-0.68 - 0.59	0.88
Medicines: provided by veterinarian rather than animal health provider	-0.11	-0.72 - 0.49	0.71	-0.13	-0.84 - 0.57	0.71	-0.3	-0.98 - 0.39	0.39
Inputs: expenditure in feed purchase and worker wages	0.29	-0.3 - 0.87	0.33	-0.25	-0.99 - 0.49	0.51	0.35	-0.29 - 0.99	0.28
Group gender : Male	0.4	-0.83 - 1.63	0.53	1.55	0.16 - 2.94	0.03	0.66	-0.63 - 1.96	0.32
Spatial thin plate spline (estimated degree of freedom)	0	NA	0.62	2.02	NA	<0.01	1.69	9	<0.01
Percentage of explained deviance – full model	18.6%								

Percentage of explained deviance – principal component regression and gender only	6% (likelihood ratio test : p- value <0.01)
Percentage of explained deviance – spatial spline and gender only	15.1% (likelihood ratio test : p-value = 0.63)

*Coef: model coefficient; CI: 95% confidence interval of the model coefficient; PV: p-value
